# Psychometric properties of a Persian version of respectful maternity care questionnaire

**DOI:** 10.1186/s12884-021-03693-w

**Published:** 2021-03-18

**Authors:** Mina Esmkhani, Masoumeh Namadian, Ali Nooroozy, Jeffrey E. Korte

**Affiliations:** 1 Social Determinants of Health Research Center, Zanjan University of Medical Sciences, Zanjan, Iran; 2 Nursing & Midwifery Faculty, Zanjan University of Medical Sciences , Zanjan, Iran; 3 Education Development Center, Zanjan University of Medical Sciences, Zanjan, Iran; 4 Department of Public Health Sciences, Medical University of South Carolina, Charleston, SC USA

**Keywords:** Respectful maternity care, Psychometric, Validity, Reliability, Birth

## Abstract

**Background:**

Providing high quality and respectful care during pregnancy and birth is one of the ways to reduce complications in women. Respectful care is a type of care that requires a valid instrument to measure. This study was conducted to determine the validity and reliability of the Persian version of the Respectful Maternity Care (RMC) questionnaire in 2018.

**Methods:**

This study was performed with 150 women (in the first 48 h after birth), who were admitted in the postpartum wards of public hospitals from 1st January until 6th April 2018 in Zanjan city in Iran. Participants were selected randomly using the Poisson distribution (Time) sampling method. After receiving permission from the questionnaire’s author, the internal consistency of the tool was measured by Cronbach’s alpha coefficient after the Forward translation of the Persian version of the tool under expert supervision. The reliability of the modified questionnaire was assessed using a test-retest method in 10 eligible postpartum women, who completed the same questionnaire again after 72 h. The validity of the tool was confirmed by exploratory and confirmatory factor analysis using LISREL and SPSS software.

**Results:**

The original RMC tool achieved an overall high internal reliability (α = 0.839). Confirmatory factor analysis of original RMC scores demonstrated poor fit indices. In LISREL proposed paths for the model, one item was excluded and a re-exploratory factor analysis was performed with the remaining 14 items. Four new subscales were defined for the revised tool including Abusive Care, Effective Care, Friendly Care, and Respectful Communication, which explained 60% of the variance.

**Conclusions:**

The revised tool included four subscales of Abusive Care, Effective Care, Friendly Care, and Respectful Communication in 14 items which explained 60% of the variance. Given the importance of providing high quality maternity care, and the variety of cultures and birth services across different countries, further research is needed on this RMC tool to evaluate its use in other countries and regions.

## Background

Pregnancy and childbirth can be considered an important time for women and their family. Despite the importance of this period, increasing maternal mortality rates and adverse consequences of pregnancy are general public health concerns. Reducing the complications of pregnancy in women and infants is one of the goals in the Third Millennium Development in which promoting prenatal care, birth and respectful care is one way to achieve this important goal [1, 2]. Providing a high-quality and respectful care during pregnancy is one of the ways to reduce the complications of pregnancy in women and newborns, as disrespectful behavior by health care providers constitutes one of the barriers to attending health care facilities and receiving professional care [2]. Respectful maternity care throughout labour and birth has been recommended in the WHO guideline for having a positive childbirth experience [3]. The absence of respectful maternity care is being increasingly recognized as a deterrent to utilization and quality of maternity care [4]. In the last two decades, women have been encouraged to give birth in health care facilities to ensure access to skilled health care professionals. However, access to quality services is not guaranteed for many women; particularly for poor women. Disrespectful and undignified care is prevalent in many countries globally, and this not only disrespects their human rights but is also a significant barrier to accessing intrapartum care services [4]. In addition, health care systems in many parts of the world, which permit the health care provider to control the birthing process, may expose obviously healthy pregnant women to unnecessary medical interventions that interfere with the physiological process of childbirth [3]. The evidence suggests that women may decline to look for care when they have previously experienced disrespectful care and mistreatment, and may also discourage others from seeking care [5, 6].

Respectful Maternity care is a kind of client-centered care, based on respect for women’s independence, expectations, authority, values, culture, and dignity. According to WHO recommendations, “Respectful maternity care refers to care organized for and provided to all women in a manner that maintains their dignity, privacy and confidentiality, ensures freedom from harm and mistreatment, and enables informed choice and continuous support during labour and childbirth” [3] This type of care is unique to each individual and his or her family, as each individual has a different family, cultural, social, and ideological background. Therefore, attention to individual differences is crucial in this type of care. According to the Human Rights Declaration, respectful care is an inalienable right of every woman [1, 2, 7–10]. The purpose of implementing respectful care is to: raise awareness and demand respectful care for clients, launch a national commitment to institutionalize maternal care as a standard of care and to mobilize communities and service providers to provide respectful care as a natural right of clients [10]. Respectful care measures are also taken to eliminate disrespect of, and abuse of clients. Several forms of abuse have been previously reported for women in hospital labour wards, including physical abuse (e.g. slapping), lack of privacy during examinations, disclosure of patients’ secrets, disrespectful care, discrimination in providing services and utilization of hospital facilities, unnecessary episiotomies without the mothers’ permission, and throwing infants on mother’s abdomen [10]. Sheferaw et al. conducted a seven-week study to determine the validity and reliability of the Respectful Care Questionnaire in postpartum women. The Cronbach alpha reliability coefficient of the tool (questionnaire) was reported as 84% [2]. Despite the importance of this issue among vulnerable groups of society and the need for this type of care in the health system, there are very few tools for evaluating and implementing respectful care in Iran, such as the study carried out by Taavoni et al. [11]. In the study by Taavoni et al., one of the original versions of the 59-item Respectful Care Questionnaire was used among women who referred to the post-partum ward of a hospital in Tehran. The present study aimed to determine the psychometric properties and localization of the RMC Respectful Care questionnaire in Iran.

## Methods

### Study aim and design

This cross-sectional analytical study (Psychometric Analysis) was conducted to determine the psychometric properties of the Persian version of the RMC Respectful Care questionnaire which was developed by Sheferaw et al. [2].

### Ethics approval

This research project received ethical approval (ZUMS.REC.1396.83) from the Vice Chancellor for Research of the Zanjan University of Medical Sciences. At the beginning of the study the purpose of the study was explained in detail for all participants, and the written informed consent of each participant was obtained.

### Study population and sampling

This cross-sectional analytical study (Psychometric Analysis) was performed with 150 women who were selected using the Poisson distribution random sampling method (Time). Eligible subjects were therefore women at any age, with adequate psychiatric health, who were admitted to the postpartum wards of the public Mousavi Hospital from 1st January until 6th April, 2018 in Zanjan city, in Iran. All eligible women who consented to participate in the study completed the questionnaire in the first 48 h after birth.

### Questionnaire/tool and translating procedures

In the present study, the original version of the Respectful Maternal Care (RMC) Questionnaire from Sheferaw et al. [2] was used after obtaining permission from the first author to translate and validate the RMC developed questionnaire in Iran. The RMC questionnaire was developed based on a review of the literature review, in-depth interviews with women who experienced labour and childbirth, followed by an expert review, measurement of face validity and content validity of the tool. A draft RMC scale with 37 items and two additional measures of global satisfaction, was administered to 509 postnatal care clients visiting facilities immediately after childbirth to 7 weeks postpartum in Ethiopia. The validity (consisting of criterion-related validity, content-related validity, and construct validity) and reliability (α = 0.845) of the RMC scale was confirmed to assess the women’s perception of respectful maternity care [2].

The RMC Questionnaire is a 15-item questionnaire, which is classified into four subscales on the basis of a 5-point Likert scale including; strongly agree (5), agree (4), don’t know (3), disagree (2), and strongly disagree (1). The questionnaire included 4 dimensions of friendly care (first 7 questions), non-discriminatory care (questions 8, 9, 10), free care (questions 11, 12, 13) ​​and timely care (questions 14, 15). Initially, the original questionnaire was translated to Persian from English (Forward translation-FWT). The translated questionnaire was then reviewed by five experts in reproductive health, health education, and epidemiology. The content validity of the translated questionnaire was determined through a review of the questionnaire by 10 experts (Reproductive Health, Health Education, and Epidemiology). The reliability (using a test-retest method) and internal consistency of the modified questionnaire was assessed in 10 eligible postpartum women, who completed the same questionnaire again after 72 h. The modified questionnaire was completed by 150 eligible women (ten participants per item). The confirmatory and exploratory factor analyses were used to assess the construct validity.

### Statistical analysis

Descriptive data analyses were performed using SPSS V.16 software to determine measures of central tendency (mean, median, and mode). To evaluate the normality of the data, the Kolmogorov–Smirnov test was used. Cronbach’s alpha co-efficient and Pearson’s Co-efficient of Correlation were used to measure internal consistency, and reliability (correlation) of modified questionnaire’s items, respectively. The confirmatory and exploratory factor analyses were used to assess the construct validity. Psychometric analysis of the tool was performed using confirmatory factor analysis and LISREL Software.

## Results

The results of the data analysis with 150 women revealed that the mean age of participants was 28.9 ± 6.2. The women’s age ranged between 15 and 43, and 86% of them were less than 35 years old. Many of the women (42.7%) and their spouses (43.3%) had not completed high school, and 27.3 and 32% of women and their spouses were illiterate, respectively. Most of the women (91.9%) were housewives, and their husbands were self-employed (39.3%). The majority of women (77.7%) reported insufficient income for living cost (Table 1). In terms of the mode of birth, 63% of women delivered by Cesarean Section (CS) and 37% had Normal Vaginal Birth (NVB).

**Table 1:** Socio-demographic characteristics of participants

Variable (N)	Groups	Frequency (%)
**Educational level**	Illiterate	41 (27.3)
(*N* = 150)	No formal qualification	64 (42.7)
	An standard high school qualification	39 (19.3)
	An university degree	16 (10.7)
**Employment**	Housewife	136 (91.9)
(*N* = 148)	Employed	12 (8.1)
**Income sufficiency**	Sufficient	7 (4.7)
(*N* = 148)	Fairly sufficient	26 (17.6)
	Insufficient	115 (77.7)
**Husband’s employment**	Public Job	23 (15.3)
(*N* = 150)	Self-employed	59 (39.3)
	Worker/ labor	28 (18.7)
	Farmer	37 (24.7)
	Unemployed	3 (2)
**Husbands’ Educational level**	Illiterate	48 (32)
(*N* = 150)	No formal qualification	65 (43.3)
	An standard high school qualification	19 (12.7)
	An university degree	18 (12)

### Internal reliability and internal consistency

The analysis of internal reliability of the original tool and related dimensions showed that the original RMC tool achieved an overall high internal consistency and reliability (α =0.839). (Table 2). With the exception of the subscale of friendly care, other subscales of the instrument did not have a good internal consistency score in the target population (Table 2).

**Table 2:** Internal reliability for the Respectful Care Questionnaire and its subscales (*N* = 150)

			Item-total correlation (r)	Cronbach’s Alpha if Item Deleted	Cronbach’s Alpha for subscales	Total Cronbach alpha
**Friendly care**						0.839 N = 150
Q.1 I felt that health workers cared for me with a kind approach			0.709	0.764	0.813	
Q.2 The health workers treated me in a friendly manner			0.644	0.777		
Q.3 The health workers talked positively about pain and relief			0.614	0.787		
Q.4 The health worker showed his/her concern and empathy			0.784	0.747		
Q.5 All health workers treated me with respect as an individual			0.552	0.795		
Q.6 The health workers spoke to me in a language that I could understand			0.343	0.825		
Q.7 The health provider called me by my name			0.276	0.830		
**Abuse- discrimination- Free Care**						
Q.8 The health worker responded to my needs whether or not I asked			0.264	0.598	0. 469	
Q.9 The health provider slapped me during delivery for different reasons			0.458	0.332		
Q.10 The health workers shouted at me because I haven’t done what I was told			0.347	0.283		
**Free Care**						
Q.11 I was kept waiting for a long time before receiving service.			0.646	−0.010	0. 580	
Q.12 I was allowed to practice cultural rituals in the facility.			−0.063	0.795		
Q.13 Service provision was delayed due to the health facilities’ internal problem.			0.675	−0.059		
**Timely Care**						
Q.14 Some of the health workers did not treat me well because of some personal attribute.			0.452	–	0.516	
Q.15 Some health workers insulted me and my companions due to my personal attributed.			0.452	–		

### Factor analysis of the tool

**Fig. 1:**
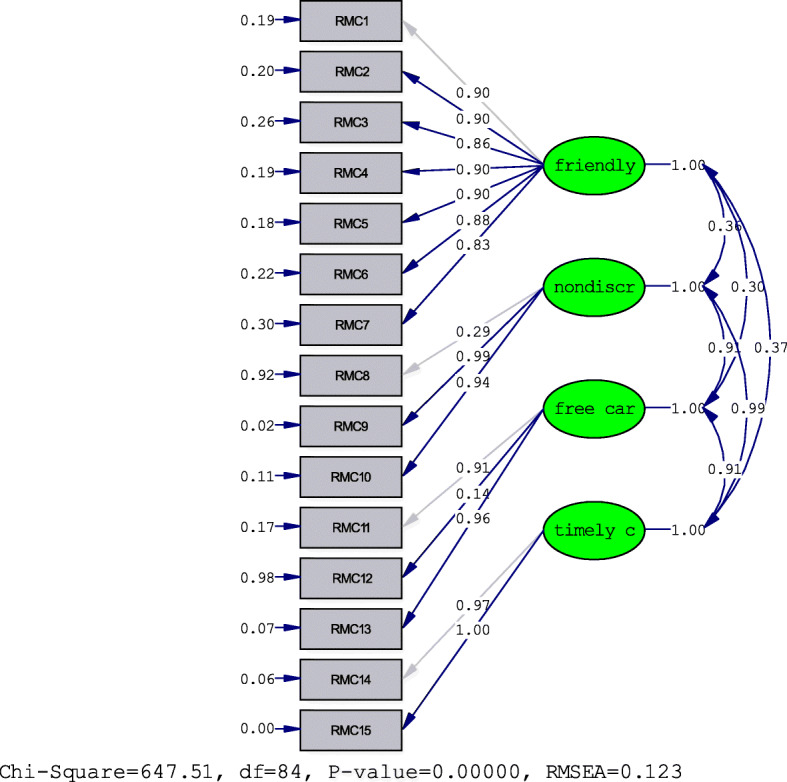
Standardized solution for the proposed domains of the respectful Maternity Care Questionnaire *Note:* All structural relationships are statistically significant (*p* < 0.01). For the sake of clarity correlations among exogenous variables and errors are not shown.

**Fig. 2:**
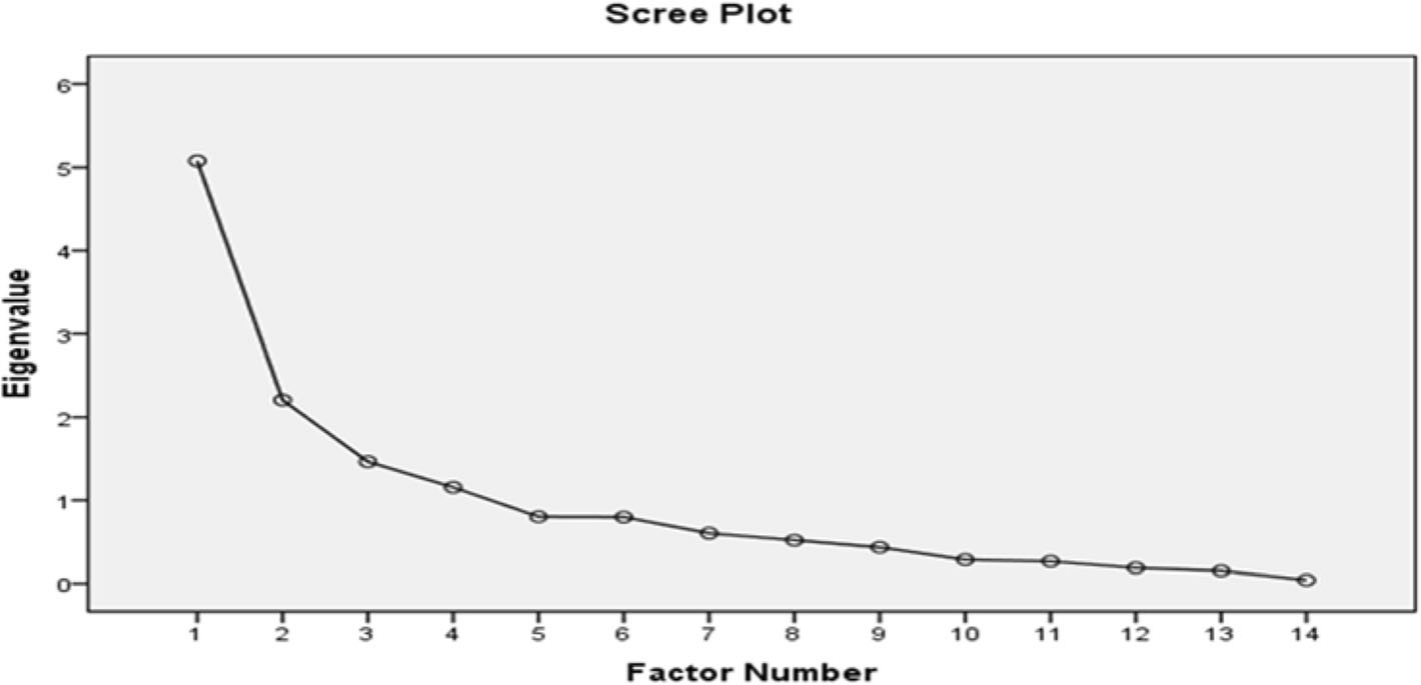
The number of Eigen Values higher than one

Factor analysis was used to analyze the questionnaire. The fitness indices obtained Chi-Square = 647/51, df = 84, *p* value < 0.0001. The results showed that the instrument did not have appropriate fitness indices (Table 3). LIZREL’s proposed paths for model correction also had little effect on the fitness indices. Therefore, the obtained data were evaluated by exploratory factor analysis. At first, the sample size and its functionality were evaluated using KMO tests, Bartlett’s test of Sphericity (Table 4). The results (Table 4) showed that the KMO was 734, indicating the suitability of the sample size. The Bartlett test was significant (*p* < 0.0001). Next, Maximum Likelihood and Varimax Rotation were used for exploratory factor analysis (Table 5). The results clarified 5 factors with Eigen values above one, (Fig. 1) (Table 6) explaining 63.84% of the variance. Examination of the Rotated Factor Matrix table showed that the Factor 5 had a factor loading only with item 7, and item 7 had no factor loading with any of the items. Since a single item could not form a scale item, it was excluded from the study and a re-exploratory factor analysis was performed with the remaining items (*N* = 14). In the 14-item instrument, 4 factors with the highest eigenvalues were extracted, explaining 60.16% of the variance. The Scree Plot (Fig. 2), Explanatory Factor Analysis, and Rotated Factor Matrix table of these factors are as follows (Tables 7 and 8). Obviously, items number 9, 10, 14, and 15 loaded heavily on the first factor, so the items were defined as Abusive Care. Items 6, 8, 11, and 13 Loaded heavily on the second factor Therefore, the above items were defined as the Effective Care dimension. Items number 3, 4, and 5 items loaded heavily on the third factor, so the above items were named as Friendly Care. Finally, items 12, 2, and 1 loaded heavily on the fourth factor, defined as the Respectful Communication dimension.

**Table 3:** The Fitting index of Original RMC Tool

Chi-square	674/51
*P* value	0/0000
df	84
RMSEA	0.123
SRMR	0.14
GFI	0.84
AGFI	0.77

**Table 4:** KMO and Bartlett’s Test

Kaiser- Mayer- Olkin Measure of sampling Adequacy	0.734
Barlte’s Test Sphericity Approx. Chi - Square	1262.405
Df	91
Sig	0.000

**Table 5:** Explanatory factor analysis for original version of RMC Tool

Factor	Initial Eigen values			Extraction sums of squared loading		
	Total	% of variance	Cumulative %	Total	% of variance	Cumulative %
RMC 1	5.144	34.293	34.293	3.375	22.499	22.499
RMC2	2.232	14.878	49.171	2.211	14.740	37.239
RMC3	1.466	9.774	58.944	1.983	13.221	50.461
RMC4	1.244	8.295	67.240	1.297	8.644	59.105
RMC5	1.082	7.215	74.455	0.711	4.740	63.844
RMC6	0. 801	5.343	79.797			
RMC7	0.771	5.138	84.935			
RMC8	0.525	3.502	88.437			
RMC9	0.434	2.895	91.332			
RMC10	0.388	2.588	93.920			
RMC11	0.286	1.905	95.826			
RMC12	0.254	1.695	97.521			
RMC13	0.187	1.247	98.768			
RMC14	0.152	1.017	99.784			
RMC15	0.032	0.216	100.00

**Table 6:** Rotated factore matrixes

	Factor^a^				
	1	2	3	4	5
RMC 9	0.971				
RMC 15	0.961				
RMC 10	0.472			0.353	
RMC 4		0.783			
RMC 3		0.699			
RMC 5		0.531	0.398		
RMC 14	0.398	0.441			
RMC 13			0.913		
RMC 11			0.722		
RMC 8			0.526		
RMC 6			0.423		
RMC 1		0.550		0.639	
RMC 2		0.451		0.626	
RMC 12				0.541	
RMC 7					0.949

Extraction Method: Maximum Likelihood

Rotation Method: Varimax with Kaiser Normalization

^a^ Rotation Converged in 6 iteration

**Table 7:** Explanatory factor analysis for Revised version of RMC Tool (14 items)

Factor	Initial Eigen values			Extraction Sums of Squared Loading		
	Total	% of Variance	Cumulative (%)	Total	% of Variance	Cumulative (%)
RMC 1	5.080	36.285	36.285	3.661	26.150	26.150
RMC2	2.206	15.754	52.039	2.778	19.840	45.990
RMC3	1.465	10.461	62.500	1.250	8.930	54.920
RMC4	1.156	8.259	70.759	.734	5.240	60.160
RMC5	.802	5.727	76.486	3.661	26.150	26.150
RMC6	.798	5.699	82.185	2.778	19.840	45.990
RMC7	.603	4.309	86.494			
RMC8	.520	3.716	90.210			
RMC9	.434	3.102	93.312			
RMC10	.286	2.046	95.358			
RMC11	.267	1.906	97.264			
RMC12	.189	1.349	98.612			
RMC13	.153	1.092	99.704			
RMC14	.041	.296	100.000

**Table 8:** Rotated factore matrix for revised version of RMC tool

	Factor^a^			
	1	2	3	4
RMC 15	0.966			
RMC 9	0.961			
RMC 10	0.479			0.392
RMC 14	0.411			
RMC 13		0.913		
RMC 11		0.725		
RMC 8		0.516		
RMC 6		0.426		
RMC 4			0.846	
RMC 3			0.683	
RMC 5		0.400	0.531	
RMC 1			0.508	0.652
RMC 2	0.356		0.417	0.647
RMC 12				0.537

Extraction Method: Maximum Likelihood

Rotation Method: Varimax with Kaiser Normalization

^a^ Rotation Converged in 5 iteration

## Discussion

Given the importance of respectful care in different communities, and the lack of studies in this field in Iran, the present study conducted to determine the validity and reliability of the Persian version of the Sheferaw’s Respectful Maternity Care. The target population in the present study was postpartum women who were transferred to the postpartum ward. One of the reasons for choosing the Sheferaw’s tool (15 items) was the low number of items used in this questionnaire, to minimize the study burden on our target population of postpartum mothers. Sheferaw conducted a study in Ethiopia on 509 postpartum women within 7 days after birth. Data were collected using a 37-item questionnaire, with an in-person interview. After analyzing the findings, the final questionnaire with 15 items was presented as a Likert scale. The reliability of this tool was assessed using Cronbach’s alpha coefficient (0.85). The results of the psychometric evaluation of Sheferaw’s tool in the present study revealed that its dimensions and related items could not be used in its original form in the study population in Iran. The use of factor analysis on the items reported that: items Number 9,10, 14, and 15 had factor loading on number one, items number 6, 8, 11, and 13 had factor loading on number two, items 3, 4, and 5 had factor loading on number 3, and items number 12, 2 and 1 had factor loading on number 4. For the present study, item No. 7 was removed as it had no effect on any of the other items, and the number of items was reduced to 14. The dimensions of the items were named as 4 dimensions: Abusive Care, Effective Care, Friendly Care, and Respectful Communication. Cronbach’s alpha for subscales was used to calculate the reliability of the four dimensions, with values of 0.757, 0.717, 0.765, and 0.710, respectively, indicating a good and acceptable Cronbach’s alpha.

A review of the literature on psychometric studies of respectful care tools revealed that despite the importance of respectful care, especially in postpartum women, this area of ​​research has received little attention from researchers in Iran. Therefore, the results of the few studies conducted in this field in Iran were compared. The only Iranian study available to the researchers in this study was the Taavoni et al. study in 2018, during which a new questionnaire of respectful care was designed, and the validity and reliability of the tool was determined. In their study, they used a questionnaire with 59 items in 7 dimensions, administered to women referred to health care centers for postpartum services. Their study showed that the QRMCQI^1^ instrument had an appropriate Cronbach’s alpha (0.93%) and the other items were within an acceptable range. Comparison of the present study with the Taavoni study clarified that the two studies were different in terms of the sample size under study and the instrument being measured. Given that the present study was designed with the primary aim of evaluating respectful care in the birth and postpartum ward, it seems that the tool used here, compared to the QRMCQI tool, as used immediately after birth, acted as revealing more accurate information compared to the results of Taavoni’s study as women become more aware of the actions taken during and after the birth, which reduces the incidence of information bias and recall bias [11]. On the other hand, due to the greater need of women for respectful care during childbirth and postpartum care (due to unnecessary and repeated examinations, lack of privacy and hormonal changes after childbirth, psychological changes in maternal and newborns), it seems that more attention should be paid to the respectful care immediately after childbirth in Iran [12–15]. Based on these principles and considering the psychological conditions and length of hospitalization of mothers, the low number of items in the relevant questionnaires, and its quickness, ease and low cost to implement, it seems that it is a useful tool. To our knowledge this is the first tool with all these criteria to be evaluated in Iran.

Another study by Rubashkin et al. in 2017 used an online survey on 501 mothers aged 18 to 45 years with an experience of natural childbirth or Cesarean section who had children aged less than 5 years. The research tool was a researcher-made questionnaire containing 111 questions in different dimensions. The results showed that the tool had a Context validity of 98%. Compared to the tool used in the present study, it had the same differences of Taavoni’s study regarding the number of items asked, the different community, the likelihood of bias, and the time taken to complete the questionnaire [16].

In the current study, confirmatory and then exploratory analyses were used to determine the construct validity of the instrument. The results of the current study are not in accordance with the results of Sheferaw’s study regarding the items of each dimension as well as the internal reliability (in the original form of the tool). One of the main reasons for this difference could be the option to select “I don’t know” at the center of the Likert scale (with score 3). In the Likert scale, the “do not know” option is expected to lead to a normal distribution and to avoid polarization of responses. There was no option for abstaining answers in the tool under study. According to AMEE GUIDE No. 87, the “No Comments” option should have been left out of the options [17]. Consideration of this in future studies can help to improve the tool. The present study is potentially the very first to validate this tool in Iran with a high sample size.

The limited generalizability of the results to the population under study is one of the limitations of this study. Our validation of this scale is only generalizable to women who attend public hospitals indicating specific socio-demographic characteristics, because the private hospitals did not agree to participate in the current study. The general health condition of participants at the time of filling the questionnaires was another limitation. Finally, another limitation of this study may have been social desirability bias stemming from women’s reluctance to truthfully answer the questions, if they were concerned that their answers could affect the quality of their health care.

## Conclusion

The revised RMC questionnaire included four subscales of Abusive Care, Effective Care, Friendly Care, and Respectful Communication in 14 items which explained a large proportion of the variance. The revised RMC questionnaire in Iran would be an appropriate tool for evaluating respectful care in women who have given birth, as it includes a low number of questions, examines four dimensions of respectful care, and has a high internal reliability in the Iranian population. This revised questionnaire therefore could be used as a valid tool in all health and medical centers to measure the level of respectful care, as one of the pillars of clients’ rights, and consequently would result in improvement of the quality of care, the services provided and the satisfaction of clients. Due to the importance of respectful care in improving the health care system and outcomes, and the limited number of relevant studies in Iran, further research in different communities are recommended.

## Data Availability

The data that support the findings of this study are available from Zanjan University of Medical Sciences but restrictions apply to the availability of these data, which were used under license for the current study, and so are not publicly available. Data are however available from the authors upon reasonable request and with permission of Zanjan University of Medical Sciences.

## Abbreviations

RMC: Respectful Maternity Care
CS: Cesarean Section
NVB: Normal Vaginal Birth

## Footnotes

1 The Quality of Respectful Maternity Care Questionnaire in Iran
